# Advanced grazing-incidence techniques for modern soft-matter materials analysis

**DOI:** 10.1107/S2052252514024178

**Published:** 2015-01-01

**Authors:** Alexander Hexemer, Peter Müller-Buschbaum

**Affiliations:** a Advanced Light Source, Lawrence Berkeley National Laboratory, 1 Cyclotron Road, Berkeley, CA 94720, USA; b Physik-Department, Lehrstuhl für Funktionelle Materialien, Technische Universität München, James-Franck-Strasse 1, 85748 Garching, Germany; c Nanosystems Initiative Munich, Schellingstrasse 4, 80799 München, Germany

**Keywords:** grazing-incidence techniques, GISAXS, GIWAXS, resonant soft X-ray scattering, GISANS, morphology, soft matter

## Abstract

The complex nano-morphology of modern soft-matter materials is successfully probed with advanced grazing-incidence techniques. Based on grazing-incidence small- and wide-angle X-ray and neutron scattering (GISAXS, GIWAXS, GISANS and GIWANS), new possibilities arise which are discussed with selected examples. Due to instrumental progress, highly interesting possibilities for local structure analysis in this material class arise from the use of micro- and nanometer-sized X-ray beams in micro- or nanofocused GISAXS and GIWAXS experiments. The feasibility of very short data acquisition times down to milliseconds creates exciting possibilities for *in situ* and *in operando* GISAXS and GIWAXS studies. Tuning the energy of GISAXS and GIWAXS in the soft X-ray regime and in time-of flight GISANS allows the tailoring of contrast conditions and thereby the probing of more complex morphologies. In addition, recent progress in software packages, useful for data analysis for advanced grazing-incidence techniques, is discussed.

## Abstract 2

Advanced grazing-incidence techniques have developed significantly during recent years. With the ongoing progress in instrumentation, novel methods have emerged which allow for an in-depth morphology characterization of modern soft-matter materials. Examples are *in situ* and *in operando* grazing-incidence small-angle X-ray scattering (GISAXS), micro- and nanofocused GISAXS, time-of-flight (TOF) grazing-incidence small-angle neutron scattering (GISANS) and surface-sensitive resonant soft X-ray scattering techniques, including the potential to investigate polarization. Progress in software for data analysis is another important aspect.

## 1. Introduction   

Soft-matter systems have received a very high level of attention in both fundamental research and applications due to their extreme diversity and their broad range of mechanical, optical, thermal, electronic or acoustic properties. Typically, soft-matter systems exhibit variable elasticity, ranging from very soft polymer chains, *e.g.* polymer brushes, to stiff colloidal particle systems (Likos, 2001 ▶). Their enormous chemical variability enables the tuning of a particularly desired functionality with high precision. Due to the different building blocks used, distinctive structures are an inherent characteristic of soft-matter systems. In the case of bulk samples, scattering techniques using X-rays or neutrons in a transmission geometry are very well established and are used to probe these micro- and nano-structures with a high degree of statistical relevance (Higgins & Stein, 1978 ▶). The most prominent techniques are small-angle X-ray and neutron scattering (SAXS and SANS) for the detection of mesoscale structures, and wide-angle X-ray and neutron scattering (WAXS and WANS) for the investigation of structures down to the molecular scale (Williams *et al.*, 1999 ▶; Fratzl, 2003 ▶). These scattering experiments are commonly used in combination with real-space imaging techniques ranging from optical microscopy to transmission electron microscopy (TEM), depending on the required resolution.

For many applications in modern soft-matter science, thin films are of tremendous interest and the need for a different approach arises. Prominent examples are thin films of conductive polymers, which attract enormous attention in organic electronics, or thin films of stimuli-responsive polymers, which receive high interest for smart coatings. In principle, thin film samples can be prepared from every soft-matter system by a large variety of different coating techniques, such as solution casting, spin coating, blade coating, dip coating, spray coating or printing. However, the morphologies in such thin films can differ strongly from the bulk structure due to a multitude of different additional effects: in the thin film geometry, interactions with both the substrate and the surrounding medium need to be considered, and these add enthalphic contributions to the free energy and thereby affect the morphology. The wall confining the film might be penetrable (*e.g.* air, gas or liquid) or might be impenetrable (solid), which affects the density profile and changes the entropy of the film. In addition, the selected film thickness itself might impose some restrictions beyond simple interactions. As a consequence, the necessity arises for a structural characterization tool for soft-matter thin films in a range of length scales similar to the bulk material. However, the reduced sample volume (thin film *versus* volume sample) causes additional difficulties, which render techniques such as SAXS, SANS, WAXS or WANS less suitable. The problem arises from the small scattering cross-section of soft-matter materials, although exceptions to this rule are very highly ordered materials such as polymer gratings of single-crystal block copolymer (Sunday *et al.*, 2013 ▶; Zhao *et al.*, 2013 ▶).

To overcome this challenge, the transmission geometry has to be replaced with a reflection geometry. Instead of an incident beam at a 90° incident angle (transmission), a very shallow incident angle α_i_ is applied. At such a shallow incident angle, the beam travels a significantly long path inside the thin film *via* the footprint effect, allowing for the required scattering interaction to occur. The beam path inside the sample scales for a sample of thickness *t* as 2*t*/sin(α_i_), where the factor 2 is due to reflection: the X-ray beam enters a film of thickness *t* and undergoes total reflection at the film–substrate interface, so combining the incoming and outgoing path results in a factor 2. Since an incident angle around the critical angle of the sample is typically used, which is well below 1° in the case of hard X-rays, such conditions are called grazing incidence, and, therefore, the related experimental techniques are grazing-incidence techniques.

In more detail, grazing-incidence small-angle X-ray and neutron scattering (GISAXS and GISANS) are the analogues of SAXS and SANS, and grazing-incidence wide-angle X-ray and neutron scattering (GIWAXS and GIWANS) are the analogues of WAXS and WANS. These grazing-incidence techniques probe the same length scales as in transmission geometry and are consequently very successfully used for structure determination in soft-matter thin films. It is worth mentioning that, for structure determination, additional scattering techniques exist such as grazing-incidence diffraction (GID), off-specular scattering and reflectivity. GID is very similar to GIWAXS and GIWANS, but does not suffer from sampling off the Ewald sphere (Dosch, 1992 ▶). In fact, GID experiments were performed quite early and pioneering work was published with descriptions such as standard X-ray diffraction in which a reflection geometry is used (Eisenberger & Marra, 1981 ▶) or X-ray diffraction under total external reflection (Dosch *et al.*, 1988 ▶). Again, hard-matter samples were studied long before soft-matter samples (Factor *et al.*, 1993 ▶). Off-specular scattering is somewhat complementary to GISAXS and GISANS because larger in-plane structures are probed at the same sample-to-detector distances (Rauscher *et al.*, 1995 ▶). Reflectivity is complementary to GISAXS and GISANS because information along the surface normal is extracted (Daillant & Gibaud, 2009 ▶).

Among the grazing-incidence techniques, those using X-rays (GISAXS and GIWAXS) were developed first. Pioneering GISAXS experiments investigated gold nano­structures on solid supports (Levine *et al.*, 1989 ▶, 1991 ▶), but the full potential of the technique was not realised and it took some time until comparable experiments on hard-matter samples were performed by other groups (Naudon & Thiaudiere, 1997 ▶; Salditt *et al.*, 1995 ▶; Thomann *et al.*, 1997 ▶). GISAXS measurements in the area of soft-matter samples were used to observe *e.g.* nano-dewetting structures of polystyrene on top of different silicon substrates (Müller-Buschbaum *et al.*, 1997 ▶). In these early experiments the name GISAXS was not well established and, as a consequence, alternative names were used to describe the scattering experiments performed, for example non-specular X-ray scattering with small-angle scattering equipment (Salditt *et al.*, 1995 ▶) or diffuse X-ray scattering under the conditions of small-angle scattering (Müller-Buschbaum *et al.*, 1997 ▶).

Perhaps due to the stronger scattering from hard-matter materials, the detectors available and the flux at synchrotrons in the 1990s, most GISAXS articles have concerned the probing of nano- and mesoscale structures from inorganic thin films. GISAXS experiments on soft-matter samples have remained scarce, although early examples included functional polymer films, such as luminescent or conductive ordered structures from nitrogen-containing polymers that formed self-organized comb-shaped supramolecules with selected amphiphilic oligomers (Knaapila *et al.*, 2001 ▶) and highly regular polyampholytic structures at silicon surfaces (Mahltig *et al.*, 2001 ▶). In addition, hybrid materials such as nano­crystalline mesoporous films prepared by evaporation-induced self-assembly (EISA) using surfactants (Gibaud *et al.*, 2003 ▶) and specially designed non-ionic block-copolymer templates (Grosso *et al.*, 2004 ▶) were successfully investigated with GISAXS. Since then, GISAXS has developed into a very popular analysis technique for soft-matter thin films (Ree, 2014 ▶), also due to the fact that it is a non-destructive method and requires no special sample preparation, except the usual deuteration in the case of neutron scattering experiments. The strong interest has inspired a variety of basic review articles on the fundamentals of the GISAXS technique [see, for example, reviews by Hamilton (2005 ▶), Müller-Buschbaum (2008 ▶) and Renaud *et al.* (2009 ▶)]. While GISAXS experiments are routinely performed at synchrotron radiation sources, the progress in laboratory X-ray source instrumentation means that GISAXS measurements have also become feasible with laboratory equipment (Altamura *et al.*, 2012 ▶; Andersen *et al.*, 2011 ▶; Siffalovic *et al.*, 2010 ▶). Thus, a further expansion of the numerous possibilities for performing GISAXS measurements will develop GISAXS into a standard characterization technique for soft-matter thin films.

More and more areas of soft-matter research require structure characterization spanning many length scales, from the atomic length scale to hundreds of nanometers. A prominent example would be the very active area of organic electronics. The combination of GISAXS and GIWAXS provides an exceptional tool for such studies, probing the molecular orientation in GIWAXS and using GISAXS up to the micron region, providing a full mesoscale approach to the problem. For this reason, a large number of device structures built up from small molecules or polymer chains have been analyzed using GISAXS and GIWAXS. Irrespective of whether the material is used in organic light-emitting diodes (OLEDs), organic field-effect transistors (OFETs), organic solar cells (OSCs) or organic photodetectors (OPDs), the morphology of the active layer is very difficult to access with real-space imaging techniques, but very successfully studied with GISAXS and GIWAXS (Chen *et al.*, 2012 ▶; Müller-Buschbaum, 2014 ▶; Rivnay *et al.*, 2012 ▶).

With the rapid revolution in instrumentation at synchrotron and neutron facilities, new possibilities have arisen in analyzing soft-matter thin films with advanced grazing-incidence scattering techniques. In the present article these new possibilities will be illustrated using a variety of selected examples from the literature. In addition to experimental progress, the development of tools and software for data reduction and analysis is of great importance for giving scientists access to advanced grazing-incidence techniques and getting the most information out of their experiments. At present, several software options have been developed which contribute significantly to a more in-depth analysis of the scattering data collected in grazing-incidence geometry (see §2.1). Progress in instrumentation has been directed, for example, to the development of narrower X-ray beams, which in turn has allowed for GISAXS and GIWAXS to be performed with such small beams and has thereby opened new possibilities in probing *e.g.* local structure information (see §2.2). With the increasing flux available at the latest generation synchrotron sources, the necessary data acquisition times have been significantly reduced, which has opened the route to *in situ* (see §2.3) and *in operando* studies (see §2.4). In parallel, a revolution in detector technology has replaced traditional CCD detectors with fast single-photon counting detectors capable of collecting hundreds of frames per second with up to 90% efficiency in the hard X-ray range. As an alternative to the hard X-ray radiation commonly used in GISAXS and GIWAXS, the use of soft X-rays and X-rays in the ‘tender’ range (1–4 keV) has made tremendous progress. Due to the absorption fine structure of the materials being probed, contrast can be shifted strongly with little change in the X-ray photon energy in the regime of tender and soft X-rays (see §2.5). An alternative probe to X-ray scattering experiments can be the use of neutrons. In GISANS (and GIWANS) the contrast conditions are very different from X-ray contrasts, which allow for new possibilities in structure determination. In particular, the combination of time-of-flight (TOF) techniques with GISANS (TOF-GISANS) is an interesting option, that enables the performance of experiments that are difficult or impossible to conduct with X-ray radiation (see §2.6).

## 2. Selected examples   

The number of experiments using advanced grazing-incidence techniques for the analysis of modern soft-matter materials has exploded during the last few years. As a consequence, it is impossible to give a comprehensive collection of experiments in this overview. Instead, we have picked selected examples from the literature to highlight new developments, in particular without making claims of being complete with respect to all recent developments.

**Figure 1:**
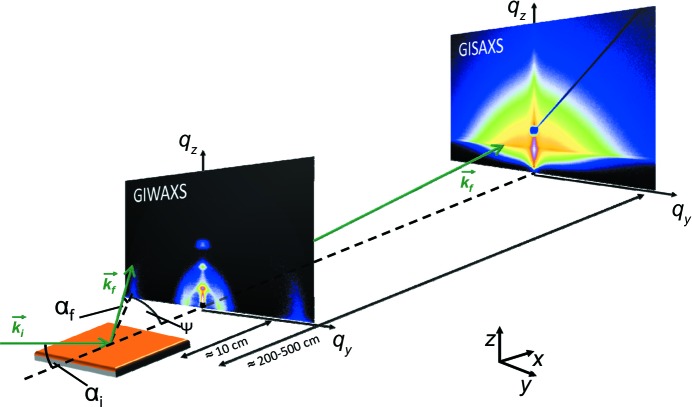
A sketch of the scattering geometry used in GISAXS and GIWAXS (GISANS and GIWANS will be analogous). The sample surface is inclined by an incident angle α_i_ with respect to the horizon. The exit angle is denoted α_f_ and the in-plane angle ψ. The color coding visualizes differences in the scattered intensity on a logarithmic scale. Typical sample-to-detector distances for GIWAXS and GISAXS are given.

A common feature of all these experiments, whether GISAXS, GISANS, GIWAXS or GIWANS, the X-ray or neutron beam impinges on the sample surface at a very shallow angle α_i_ < 1° (measured with respect to the sample surface), while the scattering is probed with a two-dimensional detector as a function of the exit angle (or out-of-plane angle) α_f_ and the in-plane angle ψ. Note that the terms ‘in-plane’ and ‘out-of-plane’ refer to the sample plane (*xy* plane) in the present article, whereas in some papers they are worded oppositely and refer to the scattering plane (*xz* plane). The coordinate system is chosen to have the X-ray beam oriented parallel to the projection of this incident beam on the sample surface. With the *z* axis oriented along the surface normal, the *y* axis is in the plane of the sample surface as well. Fig. 1 ▶ illustrates this scattering geometry. With the wavelength λ, the wave vector transfer **q** is given by
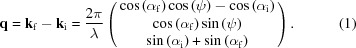
As shown in Fig. 1 ▶, typical sample-to-detector distances for GISAXS and GISANS are of the order of 130–500 cm, and for GIWAXS and GIWANS about 10–50 cm, depending on range and detector size. For small angles (in GISAXS/GISANS), the two-dimensional detector probes mainly the *q*_*y*_,*q*_*z*_ information; since *q*_*x*_ << *q*_*y*_,*q*_*z*_, the solid angle probed on the Ewald sphere is small and therefore the curvature is negligible. At large angles (in GIWAXS/GIWANS) the fixed incident angle and the use of a flat area detector cause problems compared with GID experiments because parts of the reciprocal space are inaccessible. The inner part of the reciprocal space is missed, which could be problematic for detecting sharp and higher order Bragg peaks. However, in the case of soft-matter systems, the significant disorder causes a smearing of the Bragg intensities, that results in broad peaks spreading out well into reciprocal space. As a consequence, such smeared Bragg peaks can be probed with a two-dimensional detector, and with GIWAXS (or GIWANS) important information about the orientation of crystals and crystalline spacings can be gained. However, care has to be taken in interpreting crystal orientation with respect to the surface normal in GIWAXS. For instance, for organic photovoltaics the π–π stacking peak in poly(3-hexylthiophene) (P3HT) systems along the perpendicular direction is not accessible in typical GIWAXS experiments (Baker *et al.*, 2010 ▶).

While GIWAXS/GIWANS are sensitive to the crystalline part of the soft-matter sample under investigation, with GISAXS/GISANS, larger scale structures are probed. Scattering originates from variations in the refractive index
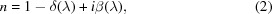
with the dispersion δ(λ) and absorption β(λ) parts for X-rays or neutrons. The sign of the imaginary part of the refractive index depends on the convention of the electric field and can be negative (Renaud *et al.*, 2009 ▶). Contrast originates from differences in the refractive indices of the materials, and contrast conditions are very different for X-rays or neutrons due to the fundamentally different scattering mechanisms. Typically, for hard X-ray scattering from soft-matter materials *n* < 1 is fulfilled, which gives rise to total external reflection of the impinging beam for angles smaller than the critical angle
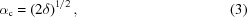
and the so-called Yoneda peak in the diffuse scattering (Yoneda, 1963 ▶), which is observed at a position α_Y_ = α_i_ + α_c_ relative to the direct beam on the detector. The factor α_i_ is due to the tilt of the sample with respect to the direct beam. It is important to note that, on the two-dimensional detector, only one point satisfies the specular condition α_i_ = α_f_, and therefore all the probed intensity is diffuse scattering. In fact, the specular peak is commonly shielded with a beamstop in order to protect the detector against the high intensity, since for α_i_ < α_c_ the direct peak has (almost) the same number of photons as the direct beam.

### 2.1. Analysis and software   

**Figure 2:**
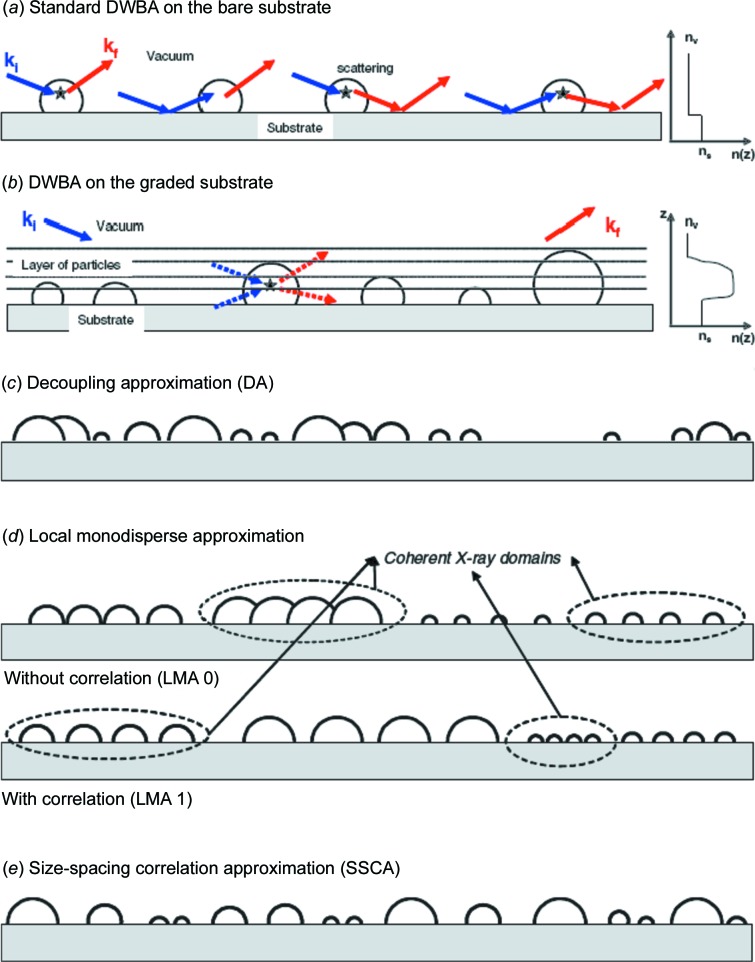
(Top) The scattering from one island along the perpendicular direction can be treated in the DWBA using, as an unperturbed state, (*a*) the bare substrate or (*b*) the graded interface and its full profile of refraction index *n*(*z*). In case (*a*), the particle form factor includes the reflection effect of the incident and scattered radiation on the substrate surface alone, as if the particle were isolated. In case (*b*), the scattering is from an upward or downward to an upward or downward propagating wave inside the particle layer. (Bottom) Schematics of the morphology corresponding to the various approximations used to calculate X-ray diffuse scattering. Particles are placed following (*c*) the DA, (*d*) the LMA, without or with correlation, and (*e*) the SSCA. Figures reprinted with permission from Lazzari, Renaud *et al.* (2007 ▶). Copyright (2007) the American Physical Society.

The diffuse scattering is typically analyzed within the framework of the distorted-wave Born approximation (DWBA) to account for the dynamic effects which occur at shallow angles close to the critical angle of the materials involved. An analysis using the DWBA is significantly more complex than an analysis of the transmission data. Nevertheless, the basic concepts from the analysis of transmission data, namely the use of form factor *F*(**q**) and structure factor *S*(**q**) contributions, are applied as well. In addition to the simple scattering event, which is well described within the framework of the Born approximation (BA), further terms in the DWBA account for reflections at the surface. The beam can be reflected before and/or after the scattering event (see Fig. 2 ▶ *a*). Without including cross-correlations (Lee, Park *et al.*, 2005 ▶; Lee, Yoon *et al.*, 2005 ▶) for a simple object located on a solid support, the initial form factor *F*(**q**) is replaced by (Rauscher *et al.*, 1999 ▶)
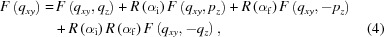
with *p*_*z*_ = (**k**_i_ + **k**_f_)_*z*_, *q*_*xy*_ = 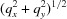 and the Fresnel reflection coefficients of the substrate *R*(α_i_) and *R*(α_f_). For larger angles α_i,f_ >> α_c_, this expression [equation (4)] simplifies into the BA, because *R*(α_i_) = *R*(α_f_) = 0. Thus, the differential cross-section for diffuse scattering in the DWBA simplifies within the effective surface approach to (Salditt *et al.*, 1995 ▶; Müller-Buschbaum, 2003 ▶)
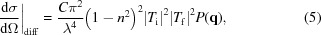
where *C* denotes the illuminated surface area, λ the wavelength used, *n* the refractive index, *T*_i_ and *T*_f_ the Fresnel transmission functions and *P*(**q**) the diffuse scattering factor, which contains the desired morphological information. Because the intensity calculated within the BA or DWBA can differ significantly (Revenant *et al.*, 2004 ▶), the use of simplifications needs to be checked carefully and will depend on the soft-matter system under investigation, as well as on the experimental setting of the GISAXS/GISANS experiment, in particular α_i_.

In the modeling of the form factor contribution, the shape and the polydispersity, so the size distribution, need to be considered (Pederson, 1977 ▶). With respect to the particle shape gained from SAXS and SANS analysis, a wide variety of different analytical expressions of form factors have been elaborated (Lazzari, 2002 ▶; Kohlbrecher, 2009 ▶). In soft-matter systems, shapes such as cylinders, spheres or lamellae are most common. The polydispersity is approximated in the so-called decoupling approximation (DA), the local monodisperse approximation (LMA) or the size-spacing correlation approximation (SSCA), as sketched in Figs. 2 ▶(*c*)–2 ▶(*e*) (Lazzari, Renaud *et al.*, 2007 ▶). In the DA it is assumed that the position and the type of scattering object are uncorrelated, which typically applies to small polydispersities (Fig. 2 ▶ *c*). In the LMA, neighboring objects are assumed to have the same shape and size, which is commonly used for polydisperse samples (Fig. 2 ▶ *d*). One has to distinguish whether the sets of monodisperse domains exist over a length scale larger than the X-ray or neutron coherence length with no correlation between size and spacing (LMA 0 in Fig. 2 ▶ *d*), or with such a correlation (LMA 1 in Fig. 2 ▶ *d*). To account for a particular correlation between particle separation and their respective sizes, the SSCA was developed (Lazzari, Leroy & Renaud, 2007 ▶).

Even for the simple case of scattering particles located on a solid support, the situation can become more complex if the particle density (*i.e.* the surface coverage) increases. In such a scenario, the incident and scattered waves are not only reflected at the flat interfaces, but also at the particle layer itself (see Fig. 2 ▶ *b*). As a consequence, this simple scenario needs to be replaced by a description within a graded interface (Lazzari, Leroy & Renaud, 2007 ▶; Jiang *et al.*, 2011 ▶). Thus, compared with SAXS/SANS, the analysis of measured GISAXS or GISANS data typically turns out to be more difficult.

It is necessary to distinguish between software used to perform the data reduction, *i.e.* to correct the data and translate the detector pixels into the correct *q* space, and software packages used for the analysis. In the case of data reduction for GISAXS (mainly to convert to *q* space and allow line cuts), four packages stand out. Developed as part of the High Data Rate Initiative in Germany, a Python-based tool called *DPDAK* with a graphical user interface (GUI) has emerged (Benecke *et al.*, 2014 ▶). This tool allows on-the-fly data reduction, line cuts and simple fitting. *NIKA* is similar, a software tool developed at the Advanced Photon Source (APS) by Jan Ilavsky that also allows on-the-fly data reduction to *q* space and GISAXS line cuts (Ilavsky, 2012 ▶). The software is free of charge, but requires the commercial *IGORPro* development package (Wavemetrics, 2008 ▶). The neutron community has developed a similar tool called *DANSE*, which is also free of charge and widely available to use (*DANSE*, 2009 ▶). In addition, a number of scientists have written their own tools. However, the list of data reduction software to perform the necessary re-meshing for GIWAXS from pixels to *q* space is very limited. Besides a variety of closed and not shared software tools, the most common tools are *GISAXSshop* (Lee, 2014 ▶) and *GIXSGUI*, a *MATLAB*-based GUI developed by the APS (Jiang, 2014 ▶) and, very recently, a Python version to perform a fully automated calibration of the experimental geometry and re-meshing on-the-fly; this effort is a collaboration between APS, the Advanced Light Source (ALS) and DESY to combine some of the features of *GIXSGUI* and *DPDAK*.

Data analysis poses a tremendous challenge with respect to complexity and approach. The list of software packages for traditional SAXS and SANS analysis is quite extensive and includes software such as *GIFT* (Bergmann *et al.*, 2000 ▶), *FISH* (Heenan, 2009 ▶), *SASFit* (Kohlbrecher, 2009 ▶), *Scatter* (Förster *et al.*, 2010 ▶), *DANSE* and tools from *Irena* (Ilavsky, 2009 ▶; Ilavsky & Jemian, 2009 ▶), to name just a few. Some simple line-scan analysis for GISAXS is included in *Scatter* and *NIKA*, but the possibilities for an in-depth analysis of GISAXS and GISANS data is quite limited. Thus, several research groups initially developed in-house analysis codes, which implemented the DWBA for simulating GISAXS/GISANS data for many different soft-matter systems (see *e.g.* Buljan *et al.*, 2012 ▶; Kim *et al.*, 2006 ▶; Modestino *et al.*, 2011 ▶; Müller-Buschbaum, 2003 ▶, 2009 ▶; Narayanan *et al.*, 2005 ▶; Park *et al.*, 2009 ▶; Rho *et al.*, 2013 ▶; Smilgies *et al.*, 2012 ▶; Stein *et al.*, 2007 ▶). Some of these tools are highly specialized to a particular topic. With the development of freely accessible dedicated software packages for the analysis of GISAXS data, such as *IsGISAXS* (Lazzari, 2002 ▶), *pyGISAXS* and *FitGISAXS* (Babonneau, 2010 ▶), the possibilities for in-depth analysis have broadened significantly. These software packages have successfully been used to model the scattering from very different systems and have found a broad usage.

Although very powerful, these software packages are constrained in the range of possible models which can be set up, that was typically triggered by the interests of their inventors. For example, *IsGISAXS* is a powerful tool for analyzing GISAXS data from nanoparticles on solid supports, with a large library of different particle shapes (from simple cylinders to complex objects such as truncated pyramids) and particle–particle correlations (including one- and two-dimensional paracyrstals, as well as more regular lattices). Beyond modeling of islands (Carbone *et al.*, 2008 ▶; Kaune *et al.*, 2009 ▶; Lazzari, Lery & Renaud, 2007 ▶; Roth *et al.*, 2003 ▶; Schwartzkopf *et al.*, 2013 ▶), *IsGISAXS* has successfully been applied to modeling porous films (Rawolle *et al.*, 2013 ▶) and polymer nanostructures (Müller-Buschbaum *et al.*, 2004 ▶). It has even progressed to the analysis of GISANS data from the active layers of organic solar cells containing conjugated polymers and small molecules (Ruderer *et al.*, 2012 ▶). However, *IsGISAXS* has limitations concerning the number of layers, inside or on top of which the nanoparticles are located. These limitations are overcome by *BornAgain* (Durniak *et al.*, 2014*a* ▶), which reproduces completely the functionality of *IsGISAXS*, but with unrestricted numbers of layers and particles and by considering diffuse reflections from layer interfaces and particles with inner structures. Moreover, with respect to GISANS, novel aspects such as neutron polarization and magnetic scattering are available in *BornAgain* (Durniak *et al.*, 2014*b* ▶).

As an alternative to the widely used software *IsGISAXS*, the package *FitGISAXS* has been developed that allows for the analysis of supported or buried objects arranged in two or three dimensions in a stratified medium. *FitGISAXS*, just like *NIKA* and *IRENA*, requires *IGORPro* as a platform. A broad variety of different systems, from buried nanoparticle layers (Babonneau *et al.*, 2012 ▶) to donor–acceptor copolymer films (Perez *et al.*, 2014 ▶) and proteins anchored on membrane surfaces (Abuillan *et al.*, 2012 ▶), have been analyzed with this package.

**Figure 3:**
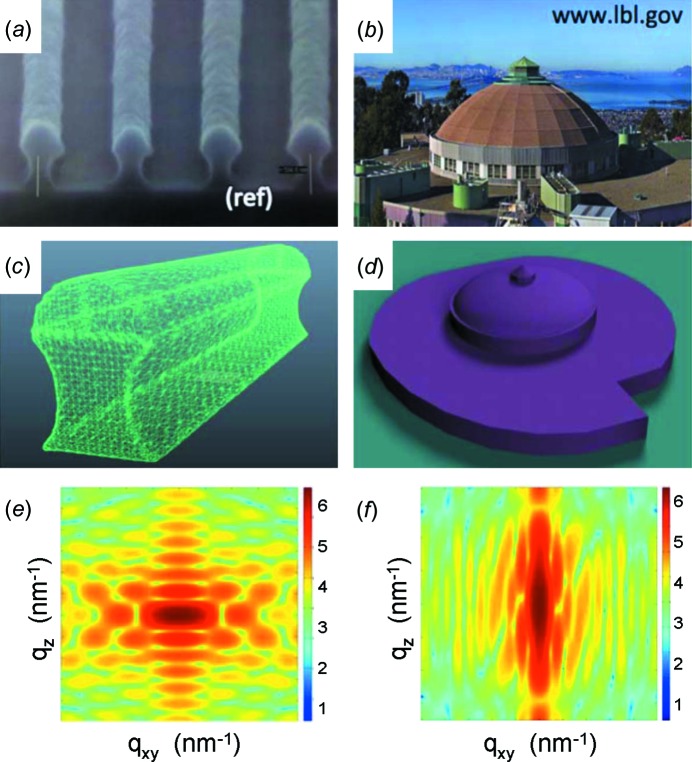
Examples of form factor calculations using *HipGISAXS*. (*a*) An SEM image of a line grating with complex side walls, (*c*) a generated discrete shape model using triangulation and (*e*) the calculated form factor. (*b*) The characteristic building of the Berkeley laboratory, (*d*) a simplified and miniaturized shape model and (*f*) the calculated form factor. The *q* range of each form factor will depend on the size of the modeled object and the degree of scattering contrast.

**Figure 4:**
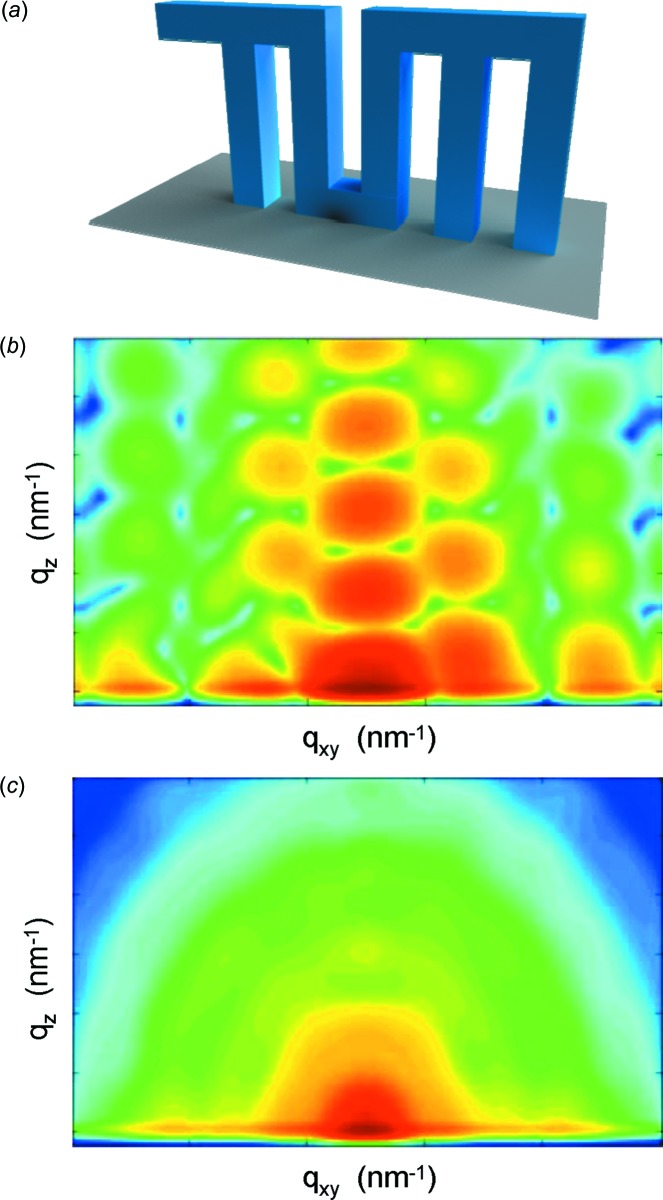
(*a*) An image of the TUM logo on a solid support as the scattering object, and computed GISAXS images, assuming only form factor contributions, using *HipGISAXS* for (*b*) a selected single-angle orientation of the object and (*c*) a radial average of orientations.

Recently, *HipGISAXS* was released (Chourou *et al.*, 2013 ▶), which goes beyond the present capabilities by using generalized sample models with a collection of custom and user-defined shaped objects, embedded in a multilayered structure. Thus, scattering objects embedded in layered films, which no longer have analytical expressions for their form factor, can be modeled, in addition to ones that do have analytical expressions for their form factor. As an example, the calculation of two form factors of complex shapes is demonstrated in Fig. 3 ▶. Neither of the selected objects, a line grating with a complex nonlinear wall shape and a miniaturized building, can be described with an analytical expression and they both deviate significantly from standard object shapes such as rectangular line gratings (Rueda *et al.*, 2012 ▶) or cylinders (Müller-Buschbaum *et al.*, 2004 ▶). In addition, the applied triangulation method enables one to choose extended objects, for example the form factor of a complex bi-continuous nanostructure, which is typical for the active layer of an organic solar cell (Rogers *et al.*, 2011 ▶; Ruderer & Müller-Buschbaum, 2011 ▶). The bi-continuous nanostructure was computed as a collection of custom shapes which were triangulated (Chourou *et al.*, 2013 ▶). The ability to compute a GISAXS scattering pattern of custom-shaped complex scattering objects is a novel feature of *HipGISAXS* (Chourou *et al.*, 2013 ▶). Fig. 4 ▶ gives the example of the calculated GISAXS pattern for a miniaturized TUM logo in the case of a selected single-angle orientation of the object (Fig. 4 ▶ *b*) and for a radial average of different orientations with respect to the incident X-ray beam (Fig. 4 ▶ *c*). In the calculation, monodisperse objects were assumed. One needs to be aware that a finer triangulation of the object surface is required in order to achieve higher accuracy in the form factor computation over a larger *q* space region (Chourou *et al.*, 2013 ▶). Thus, highly complex shapes, as exemplified by the TUM logo, can require meshes of the order of millions of elements and the corresponding GISAXS pattern calculation can be very demanding in terms of computer power. To avoid these problems, the *HipGISAXS* package was developed in a parallelized version, which runs on multiple general-purpose graphical processing units (GPGPUs) and an Intel Xeon-Phi multi CPU (Chourou *et al.*, 2013 ▶). The parallelized simulation code is well suited for performing fast simulations, which will be of growing importance due to the increasing number of two-dimensional GISAXS data sets (use of larger area pixel detectors in combination with shorter data acquisition times) collected at powerful synchrotron radiation sources.

Moving from small-angle to wide-angle scattering, several software packages are available. With *SimDiffraction*, a tool for analyzing GIWAXS data from textured thin films has been established (Breiby *et al.*, 2008 ▶), such as the crystalline structure of active layers of small-molecule solar cells (Gevaerts *et al.*, 2014 ▶), and this has been applied to porous films as well (Chavez Panduro *et al.*, 2014 ▶; Voss *et al.*, 2014 ▶). As an alternative, *GIXSGUI* has been used for simulating space groups in GIWAXS (Jiang, 2014 ▶). Thus, several different options of powerful software packages exist today, helping in the analysis of GIWAXS, GISAXS and GISANS data, which can be understood as a key to further broadening of the application of advanced grazing-incidence techniques for modern soft-matter materials analysis.

### 2.2. Micro-beam and nano-beam GISAXS   

Generally, scattering experiments are performed to obtain structural information with a high statistical relevance. In GISAXS, GISANS, GIWAXS and GIWANS the structure information is obtained from inside the illuminated film volume, which is greatly expanded by the use of a shallow incident angle due to the so-called footprint effect of the beam impinging on the sample. As a consequence, the use of large sized X-ray or neutron beams significantly increases this illuminated volume. In many GISAXS experiments, X-ray beam diameters of the order of 0.1–1 mm have been used as standard. For example, at the instrument BW4 at the HASYLAB/DESY in Hamburg, the standard beam size for GISAXS is 0.4 × 0.4 mm [full width at half-maximum (FWHM) in horizontal (H) × vertical (V) direction] (Müller-Buschbaum, 2003 ▶; Roth *et al.*, 2006 ▶). At CHESS D-line in Cornell, a typical beam size in GISAXS/GIWAXS geometry is 0.5 × 0.1 mm (Busch *et al.*, 2003 ▶, Smilgies *et al.*, 2002 ▶) and at the Austrian SAXS beamline of the synchrotron radiation facility ELETTRA, Trieste, in GISAXS/GIWAXS the common beam size is 1.0 × 0.15 mm (Amenitsch *et al.*, 1995 ▶, 1997 ▶). At the low-background-intensity focusing SAXS undulator beamline at the Australian Synchrotron, the beam size at the sample is typically 220 × 100 µm, with the beam focused at the sample position (Kirby *et al.*, 2013 ▶). At beamline I07 at the UK’s Diamond Light Source the typical beam size at the sample position is 150 × 200 µm (Arnold *et al.*, 2012 ▶). At the upgraded beamline 8-ID-E at the APS in Argonne, for most GISAXS and GIWAXS experiments, the beam size is typically set at 100 × 50 µm (Jiang *et al.*, 2012 ▶). A further reduction in beam size beyond the standard size results in a significant decrease in flux at the sample position at these instruments.

In contrast, some instruments allow for the use of smaller X-ray beams, *e.g.* ID13, the microfocus beamline at ESRF, Grenoble, which operates a microgoniometer with 5/10/30 µm beams (diameter), or microbeam optics composed of compound refractive lenses (CRLs) and a defining collimator to give a 5 µm minimum beam size, or Kirkpatrick–Baez (KB) mirror optics for a 250 nm beam (Riekel *et al.*, 2010 ▶). At beamline P03, also called the microfocus and nanofocus X-ray scattering (MiNaXS) beamline, at PETRAIII, DESY in Hamburg, operating at an energy of 12.8 ± 0.1 keV, a standard beam size of 23 × 13 µm can be achieved at the sample position by using 16 beryllium CRLs (Buffet *et al.*, 2012 ▶), and even smaller beams can be achieved by the use of an intermediate focus (Santoro *et al.*, 2014 ▶) or the nanofocus option (Krywka *et al.*, 2012 ▶).

**Figure 5:**
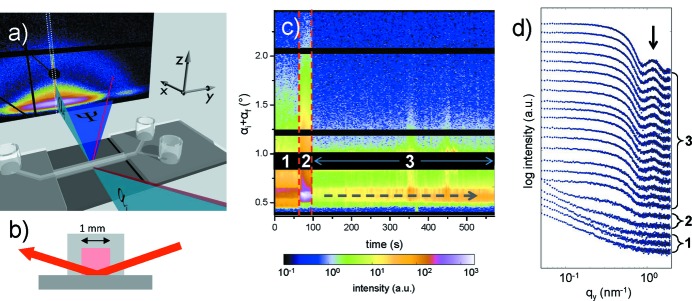
(*a*) GISAXS setup with a microfluidic cell. The incoming X-ray beam with incident angle α_i_ is depicted in red. The scattered intensity, with exit angle α_f_ and out-of-plane angle Ψ, is recorded on a two-dimensional detector. (*b*) An illustration of the X-ray beam transmitted through the channel walls and its footprint on the sample surface. Note that, for clarity, the incident angle and the X-ray beam are not to scale. (*c*) A composite image of vertical line profiles at *q*_*y*_ = 0 for 210 consecutive measurements scanning the microfluidic channel along the *y* direction while gold nanoparticles attach to a poly(ethyleneimine) thin film. Regions in black correspond to the position of the specular beamstop and the inter-module gaps of the Pilatus detector. Numbers indicate the different regimes in the microfluidic experiment. (*d*) Horizontal line profiles for an *in situ* microfluidic experiment as the sum of ten consecutive measurements. For clarity, the intensity is shifted along the intensity axis, with the initially dry film at the bottom. Reprinted with permission from Santoro *et al.* (2014 ▶). Copyright (2014) AIP Publishing.

The use of a moderately microfocused X-ray beam allows for new perspectives in GISAXS, since smaller samples can be investigated. The advantage of using small beams becomes apparent if sample preparation on a square-centimeter scale is challenging, or for samples in a complex sample environment. As an example for a complex sample environment, a combination of GISAXS with a microfluidic cell shows the great potential of this approach (Körstgens *et al.*, 2014 ▶; Santoro *et al.*, 2014 ▶). GISAXS at the solid–liquid interface typically suffers from the high absorption of the X-ray beam in the liquid phase in the common energy range for hard X-rays. With the use of a microfluidic device instead of a liquid cell, the X-ray beam path inside the liquid is significantly reduced, which allows for the use of the common X-ray energies available at beamlines typically used for GISAXS (Moulin *et al.*, 2008 ▶). Of course, the sample area inside the microfluidic cell is strongly reduced compared with a liquid cell as well, which would also result in over-illumination using a large X-ray beam. Fig. 5 ▶ shows an example using GISAXS in combination with a microfluidic cell (Santoro *et al.*, 2014 ▶). The attachment of gold nanoparticles to a poly(ethyleneimine) film surface from the flow of a gold nanoparticle solution through the microfluidic channel was monitored. Figs. 5 ▶(*a*) and 5 ▶(*b*) illustrate the scattering geometry and the combination of GISAXS and the microfluidic cell. Consecutive GISAXS measurements were performed by scanning the microfluidic channel along the *y* direction while gold nanoparticles were adsorbed at the surface. Due to the large number of collected two-dimensional GISAXS data, plotting all the two-dimensional GISAXS patterns or all corresponding line cuts from the two-dimensional GISAXS data is not meaningful. Instead, the use of so-called mappings has been established to help visualize the data better. Fig. 5 ▶(*c*) shows such a mapping composed of vertical line cuts from the two-dimensional GISAXS data, plotted as a function of time. In such a representation, changes are easily observed. However, the analysis is performed on complete two-dimensional GISAXS data or the line cuts. Fig. 5 ▶(*d*) shows a selection of horizontal line cuts to demonstrate the clear changes in the scattering data arising from lateral structures (Santoro *et al.*, 2014 ▶).

The availability of X-ray beams with a diameter of only a few micro- or nanometers offers additional new and exciting possibilities in GISAXS and GIWAXS. With a significantly smaller X-ray beam, very local scattering experiments can be performed, which makes the structure determination of inhomogeneous samples feasible. With a larger beam, the inhomogeneous morphology would be averaged and local information would be inaccessible, whereas with the use of X-ray beams with a diameter of a few micro- or nanometers the illuminated area is reduced and local structure information can be restored. Since many practical samples are intrinsically inhomogeneous within a few micrometers, for example devices and sensors, GISAXS and GIWAXS can be applied to such samples. However, one needs to note that, due to the shallow incident angle, a small beam is still elongated in the direction along the beam (*e.g.* a 5 µm beam at an incident angle of 0.975° elongates to 300 µm, and at an incident angle of 0.3° to 950 µm). As a consequence, the high resolution given by the size of the X-ray beam is only applicable in one direction (perpendicular to the incident beam), which makes one-dimensional gradient samples an interesting target. In addition, it is worth mentioning that the demand for alignment is significantly increased compared with measurements using large beams due to the need for precise location of the illuminated area. To enable positioning of the area of interest with respect to the X-ray beam, the sample position is typically equipped with a high-resolution optical microscope.

**Figure 6:**
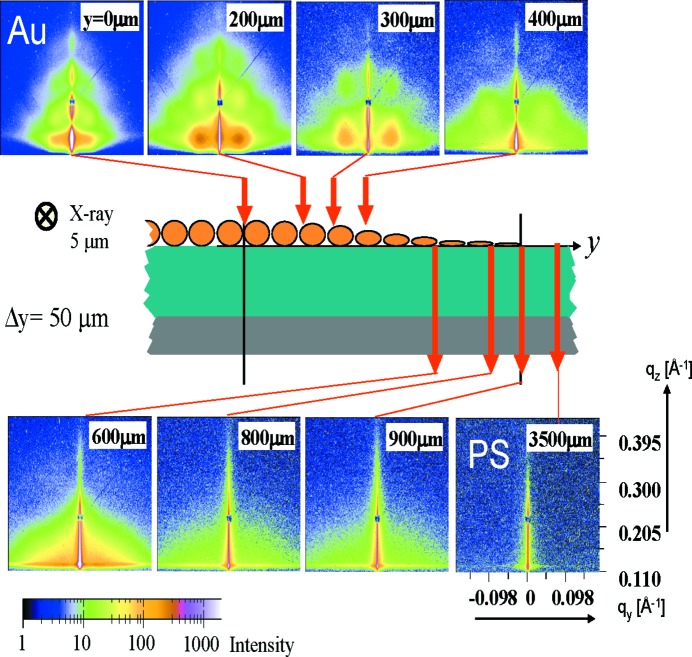
A scanning microbeam GISAXS investigation of a gold gradient on a PS film. Two-dimensional GISAXS patterns were recorded at different positions along the gradient, as illustrated for selected positions. The arrows indicate the positions of the eight patterns. The scattering pattern undergoes characteristic changes. The specular reflected beam (*q*_*z*_ = 0.22 Å^−1^) is shadowed by the rectangular beam stop. Adapted with permission from Roth *et al.* (2004 ▶). Copyright (2004) Elsevier.

Pioneering microbeam GISAXS (µ-GISAXS) measurements were performed by Müller-Buschbaum, Roth and co-workers at the ID13 beamline at ESRF (Müller-Buschbaum, Roth *et al.*, 2003 ▶; Roth *et al.*, 2003 ▶). As model systems, a two-step dewetted polystyrene (PS) film, which exhibits a nano-dewetting and a micro-dewetting structure, was investigated (Müller-Buschbaum, Roth *et al.*, 2003 ▶) and a gold cluster gradient on top of a PS film was analyzed (Roth *et al.*, 2003 ▶). Both experiments used an X-ray beam of 5 µm diameter and both scanned the sample with respect to the fixed beam position. The applied optics allowed the resolution of structures as small as 100 nm, which demonstrates the low beam divergence due to the applied X-ray optics. Driven by this success, the use of µ-GISAXS was extended to gradient multilayers of self-assembled nanometer-sized noble metal clusters on top of polymer layers (Roth *et al.*, 2004 ▶), thin cellulose layers prepared by the Langmuir–Blodgett technique (Roth *et al.*, 2005 ▶), highly ordered monolayers of polymethylmethacrylate (PMMA) beads (Frömsdorf *et al.*, 2006 ▶), Langmuir–Blodgett protein films (Pechkova *et al.*, 2009 ▶), linear aliphatic polyester coatings prepared by spin-coating (Hernández *et al.*, 2010 ▶), surface-grafted two-dimensional PS/polyvinylmethylether (PVME) blend films on nano-mechanical cantilever sensor (NCS) arrays (Lenz *et al.*, 2010 ▶), and stimuli-responsive diblock copolymer brushes composed of poly(2-hydroxyethyl methacrylate) as the bottom block and poly(*N*-isopropylacrylamide) as the top block on the surface of silicone rubber (Jalili *et al.*, 2013 ▶). All these different samples would not have allowed for meaningful GISAXS measurements without the use of micrometer-sized X-ray beams. As a selected example, Fig. 6 ▶ shows the investigation of a gold nanoparticle gradient on a PS film deposited by thermal evaporation (Roth *et al.*, 2004 ▶). The gradient was established by the use of a shadow mask, which is a frequently applied technique in many device fabrication routes (*e.g.* in organic electronics) to create contacts of a defined size. The edge of the gold contact exhibits a gradient, which was probed to follow the evolution of gold nanostructures beyond the optically accessible limit. The one-dimensional gold gradient was scanned in steps of 50 µm by shifting the sample perpendicular to the X-ray beam, using an X-ray beam of 5 µm diameter. At each position an individual GISAXS measurement was performed, which resulted in local structure information, as can be seen from the differences in the two-dimensional GISAXS patterns in Fig. 6 ▶. In total, a range of 3500 µm was scanned and the GISAXS data were modeled using *IsGISAXS*. The extracted structure information was compared with the optical behavior of the gold nanoparticles. These show well defined plasmon resonances (Roth *et al.*, 2004 ▶), which are of great relevance for determining pixel sizes in organic electronics.

With continued progress in beamline optics, further reductions in beam sizes have been realized, enabling the use of X-ray beams below 1 µm diameter in so called sub-micrometer GISAXS (sub-µ-GISAXS) experiments. In the initial experiments, a beam diameter of 0.9 µm was used to analyze local defects, such as cracks in blends of PS and poly(*n*-butyl­acrylate) (PnBA) (Müller-Buschbaum *et al.*, 2005 ▶), flow-induced phase separation structures in blends of PS and PnBA (Müller-Buschbaum *et al.*, 2006 ▶), local scale structures in blended films (Müller-Buschbaum *et al.*, 2007 ▶) or gradients in colloidal thin gold films deposited from aqueous solution (Roth, Müller-Buschbaum *et al.*, 2007 ▶). With a further reduced beam diameter of 500 nm, the systems under investigation have included conjugated coil–rod copolymers, such as the diblock copolymer polystyrene-*block*-poly(*p*-phenylene) (Al-Hussein *et al.*, 2009 ▶), and the local gold contact morphology on a photoactive polymer film, which is a material combination of interest for devices in organic electronics (Ruderer *et al.*, 2010 ▶).

So far, the minimum demonstrated X-ray beam size in GISAXS experiments is 300 nm and the corresponding experiments are named nanobeam GISAXS (nGISAXS). With a beam diameter of 300 nm, GISAXS measurements have been performed on ordered nanoparticles at the air–water–substrate boundary in colloidal solutions (Roth, Autenrieth *et al.*, 2007 ▶) and solution-cast gradients consisting of colloidal gold nanoparticles on top of a silicon substrate (Roth *et al.*, 2010 ▶). The use of a high-brilliance 300 nm X-ray beam allows for acquisition times of 120 s per two-dimensional GISAXS pattern and this has enabled *in situ* investigations such as the nanoscale structuring that occurs during evaporation of water (Roth, Autenrieth *et al.*, 2007 ▶). With this approach, the transfer of lateral order and vertical layering were identified at the three-phase air–solution–substrate contact line as a function of time.

Since progress in X-ray optics is ongoing and more and more beamlines at synchrotron radiation facilities offer nanometer-sized X-ray beams, the use of nGISAXS can be expected to grow strongly. Of course, the demand for sample alignment increases with decreasing beam size, and the resolvable range of length scales decreases with decreasing beam diameter as well. Due to the use of high-brilliance X-ray beams, the potential for inducing radiation damage becomes an important aspect and careful tests with respect to potential radiation damage need to be performed. Moreover, the coherence of the X-rays needs to be taken into account. The micro- and nanobeam experiments discussed above did not make full use of beam coherence. Under coherent conditions, the GISAXS or GIWAXS data will be perturbed by a speckle pattern, which makes the common analysis discussed above difficult. However, the use of coherent X-rays in GISAXS and GIWAXS geometry enables new possibilities. It allows the analysis of temporal correlations of the scattered intensity and thereby not only gives access to ensemble averaged properties, but also enables probing of the dynamics of the system (Vartanyants & Robinson, 2003 ▶; Yefanov *et al.*, 2009 ▶). However, in the case of soft-matter systems, the high photon flux typically imposes serious problems with radiation damage in the samples, which can affect the dynamics and, in more severe cases, the static properties (the morphology) as well. As a consequence, coherent GISAXS experiments are very rare, particularly for soft-matter samples. As a recent example, the erosion of a GaSb surface by ion-beam sputtering was probed with GISAXS using coherent X-rays. The process turned out to be age-dependent and slowed down with sputtering time (Bikondoa *et al.*, 2013 ▶). However, with the advent of diffraction-limited sources in the near future we can expect the field of coherent GISAXS to grow very strongly. To date, only a very limited part of the real potential offered by the use of coherence has been explored, *e.g.* in a recent experiment coherent X-rays were used in a reflection geometry to recover the real-space image (Roy *et al.*, 2011 ▶).

### 2.3. *In situ* GISAXS and GIWAXS   

Instead of the dynamic properties of soft-matter samples, so far the research focus has put more emphasis on kinetic properties. Kinetic information, meaning the time-dependent evolution of characteristic length scales, has received strong attention and is typically addressed with *in situ* GISAXS and GIWAXS measurements. In general, *in situ* GISAXS and GIWAXS experiments are not limited to small X-ray beams and are successfully performed with a wide variety of different beam sizes. Among the numerous *in situ* GISAXS and GIWAXS experiments, one main area of study is to follow the evolution of structure during film preparation. In such experiments, the soft-matter film is applied using techniques such as drop casting, doctor blading, spray coating, spin coating, printing, vacuum deposition or other deposition methods and the scattering experiment is performed *in situ* during the deposition. Another exciting area of research is to follow structural transformations in soft-matter films during a post-production treatment. The most common post-production treatments are thermal and/or vapor annealing.

Following structure evolution during deposition is demanding due to the fast processes involved, which in turn require very short data acquisition times. Moreover, special equipment needs to be developed, as most commercial coaters cannot be easily integrated into a synchrotron radiation instrument. With the ongoing progress in instrumentation development, very short counting times (of the order of milliseconds) are possible today and several custom-made coaters have been successfully operated *in situ* with GISAXS and GIWAXS at a synchrotron (Richter *et al.*, 2014 ▶). As an example of kinetics with moderate time constants, the composition dependence of the structural evolution during drying of doctor-bladed model films for organic solar cells was probed. The selected model system consisted of blends of poly(3-hexylthiophene) (P3HT) and [6,6]-phenyl C_61_-butyric acid methyl ester (PCBM) with different blend ratios (Sanyal *et al.*, 2011 ▶). GIWAXS data were collected for 3 s each at intervals of 50 s as the dichlorobenzene solvent evaporated from the doctor-bladed wet film, thereby enabling an observation of the microstructure evolution in real time during blend crystallization. The results obtained provided a microscopic understanding of the composition dependence of the film formation from solution, which is valuable information for morphology optimization guided from fundamental understanding. The evolution of morphology during solvent evaporation of cast blend films of a diketo­pyrrolo­pyrrole-based low bandgap polymer (pDPP) with PCBM is a second example (Liu *et al.*, 2012 ▶). For different solvents and solvent mixtures (*e.g.* dichlorobenzene and chloroform), *in situ* GIXD and GISAXS experiments were performed during solvent evaporation. The kinetics were slow, as the changes in structure progressed over more than 2000 s.

In addition to polymer film deposition, small-molecule deposition has been studied as well. For example, the nucleation and growth of 6,13-bis(tri-isopropylsilylethynyl) (TIPS)-pentacene was probed with *in situ* GIWAXS during drop casting (Li *et al.*, 2013 ▶) or blade coating (Smilgies *et al.*, 2013 ▶). In the case of drop casting, heterogeneous nucleation and the sustained surface growth of a highly aligned lamellar structure at the solid–liquid interface were observed, at the expense of homogeneous nucleation (Li *et al.*, 2013 ▶). Early experiments on the self-assembly process of surfactant template thin films using GISAXS were performed by Doshi *et al.* (2003 ▶). In the case of blade coating, high-speed solution shearing, in which a coating knife spreads a drop of dissolved material onto the substrate, was investigated. The impact of solution shearing and the subsequent drying and crystallization of a thin film of conjugated molecules were probed (Smilgies *et al.*, 2013 ▶). While solvent evaporation is slower for a solution-cast film, in the case of blade coating, the kinetics are typically faster due to the lower solvent volume which needs to evaporate.

Among the fast wet-chemical deposition methods, spray coating and spin coating have received the most attention so far. For example, the structural evolution of gold nano-domains by spray deposition on thin conductive P3HT films has been probed with *in situ* GISAXS (Al-Hussein *et al.*, 2013 ▶). Gold on P3HT is a frequently applied material combination for organic electronics such as organic solar cells. During the *in situ* investigation of the spray deposition, GISAXS data were collected with a 20 ms counting time for each two-dimensional GISAXS data set, since the entire spray process lasts only a few seconds. Thus, compared with the kinetics addressed in solution casting or blade coating, completely different kinetics were addressed here. Of course, such experiments demand a very high flux and fast detectors to allow the extremely short data acquisition times to be feasible. Similar demands are true for investigations related to *in situ* spin coating, as solvents such as chlorobenzene are fully evaporated in the first 4 s during spinning under ambient conditions. Direct observation of photoactive layer formation, as it occurs during spin-coating, was recently realized *via* a combination of GISAXS and GIWAXS (Chou *et al.*, 2013 ▶). With a frame rate of 10 Hz, the spin coating of a P3HT:PCBM solution was followed, thereby providing internal structural and morphological information during the drying process. The final thickness of the P3HT:PCBM layer was reached after 5.5 s, whereas crystallization occurred much earlier.

High-speed *in situ* investigations are not limited to polymer film deposition. Late-stage crystallization and healing in the case of small-molecule films relevant for organic thin film transistor (OTFT) applications, such as TIPS-pentacene, have also been followed with GIWAXS *in situ* during spin-coating (Chou *et al.*, 2014 ▶). Due to the rapid quenching which is characteristic of spin coating, a technique three orders of magnitude faster than drop-casting, the crystallization differs substantially from that observed in the case of slower deposition methods.

While the *in situ* investigation of film deposition is quite rarely studied, *in situ* studies of morphological transitions are quite common. Again, special equipment is required, such as solvent vapor annealing chambers or heat plates. However, the typical time constants involved in morphological transitions are significantly larger than those governing film deposition, which places fewer demands on the time resolution of the *in situ* scattering experiment. As a consequence, longer data acquisition times still allow the following changes in the characteristic length scales. Thus, these types of experiments were realized much earlier. For example, the evolution of nanopores during the thin film formation of porous dielectrics from composite films of a soluble poly(methylsilsesquioxane) precursor and a four-armed poly(∊-caprolactone) was studied with *in situ* GISAXS (Lee, Yoon *et al.*, 2005 ▶).

Among the systems studied with respect to kinetic structure transformations, diblock copolymer films have received the most attention. Compared with blend films, such as the frequently studied P3HT:PCBM system, diblock copolymer films exhibit significantly better ordered structures due to micro-phase separation [for reviews, see Fasolka & Mayes (2001 ▶), Hamley (2003 ▶), Li *et al.* (2005 ▶) and Luo & Epps (2013 ▶)]. Thus, the GISAXS patterns show characteristic Bragg peaks arising from the order in the micro-phase separation structure. Moreover, order–order transitions between different ordered structures are also possible. Using *in situ* GISAXS, the transitions of the micro-phase separation structure has been observed for thin films of an asymmetric polystyrene-*block*-polyisoprene diblock copolymer, together with a shift in the transition temperatures compared with bulk samples (Shin *et al.*, 2009 ▶). For diblock copolymer films of poly(styrene-*block*-4-vinylpyridine), a solvent vapor-induced orientation switching was found with *in situ* GISAXS (Gowd *et al.*, 2010 ▶). The reactive annealing of poly(*tert*-butyl meth­acrylate)-*block*-poly(methyl methacrylate) diblock copolymer thin films *via* solvent and heat into poly(methacrylic acid)-*block*-poly(methyl methacrylate) was also followed with *in situ* GISAXS (Sun *et al.*, 2011 ▶). In the case of the asymmetric diblock copolymer polystyrene-*block*-poly(4-vinylpyridine) in thin films exposed to tetrahydrofuran (THF) vapor, which is a selective solvent for the majority PS block, a significant narrowing of the size distribution of the cylindrical microdomains and of the center-to-center distance distribution between them was seen using *in situ* GISAXS experiments (Park *et al.*, 2009 ▶).

**Figure 7:**
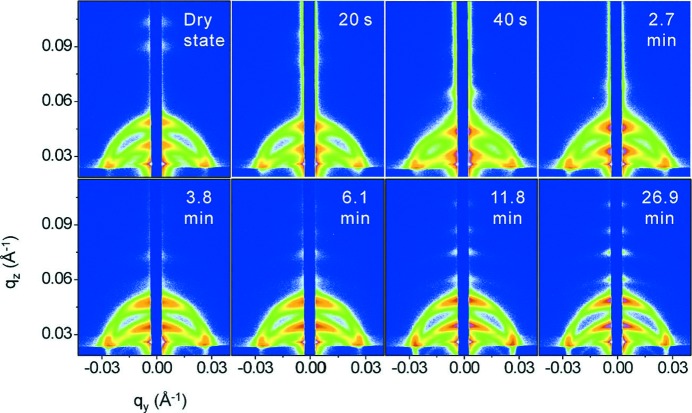
Two-dimensional GISAXS data of a lamellar poly(styrene-*b*-butadiene) diblock copolymer film during the first swelling step at the times given. The logarithmic intensity scale runs from 10 to 7000 counts for all images. Reprinted with permission from Di *et al.* (2012 ▶). Copyright (2012) American Chemical Society.

Similarly, *in situ* GISAXS was used to monitor the real-time changes occurring within films of a cylinder-forming poly(α-methylstyrene-*block*-4-hydroxy­styrene) diblock copolymer as they were swollen in THF and acetone solvent vapor (Paik *et al.*, 2010 ▶). In the case of lamellar poly(styrene-*block*-butadiene) diblock copolymers, the formation of additional parallel lamellae was measured during treatment with saturated cyclohexane vapor, a solvent slightly selective for polybutadiene. The formation of additional parallel lamellae was attributed to the increased degree of coiling (Di *et al.*, 2010 ▶). A stepwise swelling of thicker films of the same co­polymer resembled qualitatively the same behavior as was found in saturated cyclohexane (Di *et al.*, 2012 ▶). During the first swelling step, a strong increase in the film thickness was monitored with white-light interferometry (WLI) and the two-dimensional GISAXS patterns showed strong changes, as shown in Fig. 7 ▶. As described by Di *et al.* (2012 ▶), in the initial stages (during the first 1.2 min), the first-order diffuse Debye–Scherrer rings (DDSRs) and the diffuse Bragg sheets (DBSs) moved to significantly lower *q* values, as expected for swelling lamellae. Afterwards, both the DDSRs and the DBSs moved back to higher *q* values. Along with the peak position movements, the intensity distribution along the DDSRs changed as well. Initially, the intensity near *q_y_* = 0 was strongly enhanced, but this maximum extended more and more toward higher *q_y_* values after 2.7 min and later bent downward along the DDSRs. These changes were accompanied by the transient broadening of the DDSRs along *q_z_* between 0.8 to 2.4 min and the transient disappearance of the third-order DDSRs and DBSs during the time range of 7 s to 6.1 min. As a consequence, the lamellar correlation broke up and additional lamellae formed, in accordance with observations in saturated toluene or cyclohexane vapor (Di *et al.*, 2010 ▶).

Using a selective solvent to trigger the reconstruction of the cylinder-forming diblock copolymer polystyrene-*block*-poly(methyl methacrylate), the minor component block was drawn to the surface and monitored using GISAXS (Xu *et al.*, 2004 ▶). Control of the lamellar microdomain orientation was reported for polystyrene-*block*-poly(methyl methacrylate) diblock copolymer films exposed to a solvent-vapor annealing process (Choi *et al.*, 2014 ▶). In this case, a substrate-independent orientation was achieved *via* use of a neutral solvent and thermal annealing. Using a densely cross-linked polymer network, designed as an organic hard mask (HM) for lithographic fabrications, a solvent-driven ordering was successfully introduced (Stenbock-Fermor *et al.*, 2014 ▶). Swelling of polystyrene-*block*-poly(2-vinylpyridine) was probed with *in situ* GISAXS as a function of time during annealing in THF, which is a neutral solvent vapor for the studied blocks (Gu *et al.*, 2014 ▶). Solvent-vapor annealing with soft shear, realized by physical adherence of a cross-linked poly(dimethyl­siloxane) to the block copolymer film during solvent-vapor annealing, has been shown to be a simple method for generating unidirectional alignment of the cylindrical domains of polystyrene-*block*-polyisoprene-*block*-polystyrene films (Qiang *et al.*, 2014 ▶). Again, structural insights were gained using *in situ* GISAXS experiments.

In addition to pure block copolymer films, hybrid films composed of block copolymers and inorganic materials have been intensively investigated as well. Using *in situ* GISAXS, Abul Kashem *et al.* (2011 ▶) followed the formation of an ordered array of metal nanoclusters. This was realized *via* selective metal oxide doping of the micro-phase separated nanodomains in thin diblock copolymer films of polystyrene-*block*-poly(methyl methacrylate).

### 2.4. *In operando* GISAXS and GIWAXS   

In contrast with *in situ* scattering studies, *in operando* scattering investigations are less well established. For an *in operando* GISAXS or GIWAXS study, a functioning element, such as a catalyst, device or sensor, together with all the necessary instrumental environment, needs to be incorporated into a beamline at a synchrotron radiation facility. For example, with *in operando* GISAXS/GIWAXS of a catalyst, the structure characterization of the materials undergoing reaction is coupled simultaneously with measurements of their catalytic activity and selectivity. Such measurements will help to gain insights into the functionality of the catalyst. *In operando* GISAXS/GIWAXS of a catalyst requires measurement of the catalyst under (ideally) real working conditions, involving comparable temperature and pressure environments to those of industrially catalyzed reactions, but with a cell modification to allow for the scattering experiment. The conditions required for the sample environment can be very challenging, ranging from harsh chemical solvents to extreme temperature and high pressure. Therefore, *in operando* studies are challenging for cell design, since the special needs of the GISAXS or GIWAXS experiments (*e.g.* X-ray transparent window positions and sizes, free beam path to the sample, reagent and product flow rates) need to be accommodated while maintaining functionality. In parallel with the scattering experiment, the parameters of the reaction need to be monitored continuously during the reaction using additional appropriate instrumentation. Thus, the complexity of the instrumentation and of the total experiment for *in operando* studies is much higher than for *in situ* investigations. Nevertheless, *in operando* GISAXS and GIWAXS studies will increase significantly in the future, given the success of *in operando* spectroscopy investigations (Weckhuysen, 2003 ▶).

**Figure 8:**
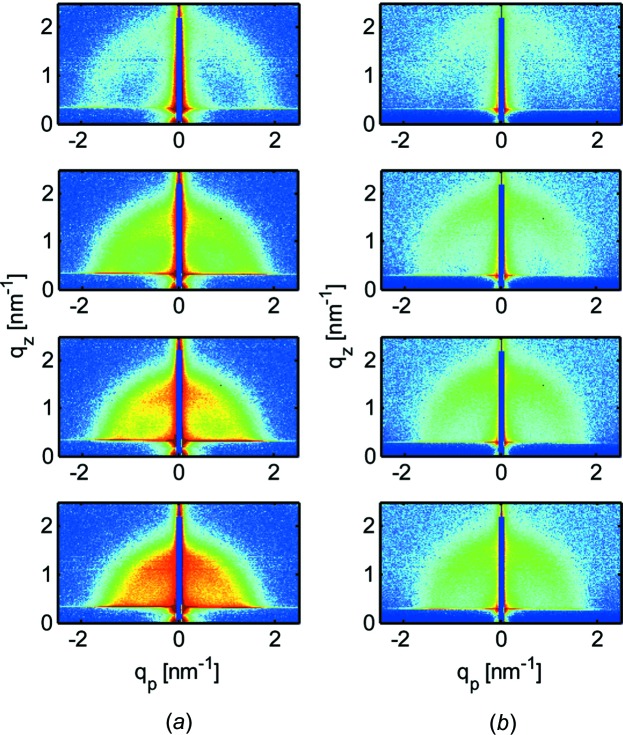
Transient GISAXS patterns of Nafion thin films during water absorption after placement in a fully saturated water-vapor atmosphere from ambient conditions. Scattering patterns (top to bottom) were collected after 3, 16, 31 and 46 min of exposure to the saturated water-vapor environment. The films were cast on (*a*) Si substrates and (*b*) OTS passivated substrates. The patterns show the swelling of the ionomer domains during water uptake, as shown by the inward shift of the ionomer scattering ring. Reprinted with permission from Modestino *et al.* (2012 ▶). Copyright (2012) American Chemical Society.

In several articles, authors have used the term *in situ* in their description of the scattering experiments performed, but the experiments conducted are, in essence, *in operando*. In particular, *in operando* studies of energy-related materials are receiving increasing attention, although frequently addressing hard condensed matter. For example, for the case of Au(001) homoepitaxial growth in Cl-containing electrolytes, the kinetics of roughening were studied with *in operando* GISAXS during electrodeposition (Ruge *et al.*, 2014 ▶). An example from soft matter deals with proton-conducting ionomers. The proton-conducting ionomer Nafion, which is widely used for electrochemical applications including fuel-cell devices, flow batteries and solar fuel generators, was studied with GISAXS under operating conditions and confined to a thin-film geometry (Modestino *et al.*, 2012 ▶). Fig. 8 ▶ shows GISAXS patterns of Nafion thin films during water absorption after placement in a fully saturated water-vapor atmosphere. After different exposure times to the saturated water-vapor environment, the GISAXS patterns change. The swelling of the ionomer domains during water uptake is shown in the inward shift of the ionomer scattering ring (Modestino *et al.*, 2012 ▶). As a consequence, the wetting interaction at thin-film interfaces can drastically affect the internal morphology of ionomers and in turn modify their transport properties.

A second example is the investigation of an organic solar cell *in operando* with GISAXS (Schaffer *et al.*, 2013 ▶). For this purpose, a special sample chamber was constructed, which allowed the probing of the current–voltage characteristics under illumination with an AM1.5 solar spectrum, simultaneously with the GISAXS measurement. A model solar cell of P3HT:PCBM was studied. During the operation of the solar cell, ageing occurred in the film. From the analysis of the GISAXS data, a change in the morphology of the active layer was found, which the authors showed to be responsible for the decrease in the short-circuit current of the device (Schaffer *et al.*, 2013 ▶). Thus, for the first time, structural ageing in organic solar cells was found as one of the mechanisms involved in the degradation of the device.

Although demanding, in the future the number of *in operando* studies can be foreseen to increase significantly, basically driven by greatly expanding areas such as energy-related materials.

### 2.5. GISAXS in the soft X-ray regime   

Traditionally, hard X-ray radiation has been used in most GISAXS and GIWAXS experiments, with a typical X-ray photon energy on the order of 10 keV, which is the common X-ray energy for SAXS, WAXS and X-ray reflectivity (XRR) experiments as well. With a change from hard to soft X-rays, meaning a decrease in the X-ray photon energy from 10 keV to lower values of the order of 100 eV, new possibilities arise. To perform GISAXS or GIWAXS in the soft X-ray regime, the experiments need to be performed at other instruments and other requirements arise. For example, soft X-rays exhibit a high absorption in air as well as in the materials being studied, which puts a strong demand for vacuum setups with no windows separating the sample and detector (Wernecke *et al.*, 2014 ▶). Moreover, the change in X-ray photon energy changes the wavelength and affects all involved angles. The incident, exit and in-plane angles typically used in the hard X-ray regime change to larger values in the case of soft X-rays and other types of calibration samples might become necessary (Wernecke *et al.*, 2012 ▶). In turn, typical sample-to-detector distances need to be adapted and are significantly shorter for soft X-rays.

**Figure 9:**
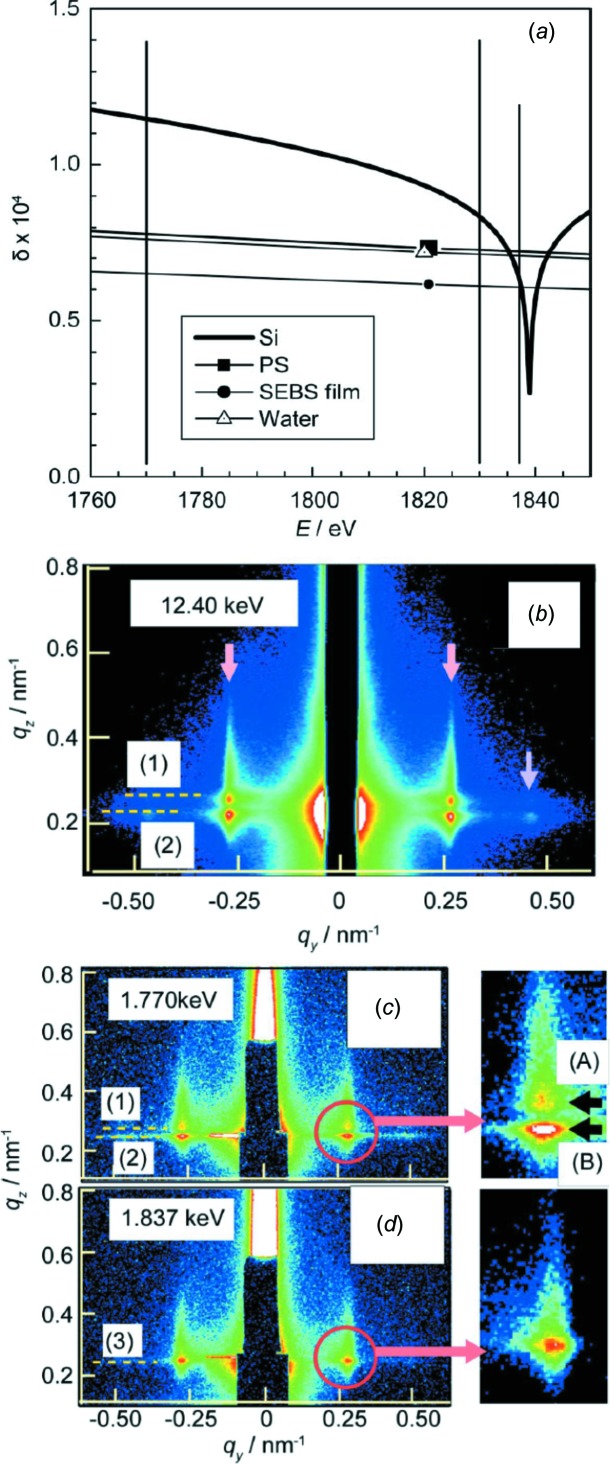
Experiments at the Si *K* absorption edge. (*a*) The real parts of the refractive indices for an Si substrate, PS, SEBS and water at the Si *K* absorption edge, as calculated from reported values of the anomalous scattering factors and densities. For SEBS, the density was determined from the critical-angle measurements with Cu *K*_α1_ radiation. The vertical lines mark X-ray energies of 1.770, 1.830 and 1.837 keV. The bottom part of the figure shows GISAXS patterns measured with X-rays with photon energies of (*b*) 12.4 keV, (*c*) 1.770 keV and (*d*) 1.837 keV. The positions marked by broken lines correspond to the calculated positions of the critical angle for (1) Si, (2) SEBS and (3) both. Adapted from Okuda *et al.* (2012 ▶).

The main idea of using X-ray radiation with smaller energies is to work at the absorption edge, which strongly affects the real and complex parts of the refractive index (Okuda *et al.*, 2009 ▶; Yamamoto *et al.*, 2014 ▶). In order to avoid strong absorption, it is common to work in an energy regime just before the absorption edge or resonant peak, where β, the absorption part of the refractive index, is very small. For example, in the case of soft-matter films on top of a silicon (Si) substrate, which is a frequently chosen substrate due to its low surface roughness, it can be interesting to work at the Si *K* absorption edge (1.840 keV) and make use of the large anomalous dispersion effect (Okuda *et al.*, 2011 ▶). At the *K* absorption edge, the real part of the Si refractive index drops strongly and can even become smaller than that of a soft-matter film on top of the Si substrate (see Fig. 9 ▶ *a*). In principle, this might even offer the possibility of contrast matching between the Si substrate and the soft-matter film or with a surrounding medium such as water (Okuda *et al.*, 2012 ▶). However, due to the very likely differences in the imaginary parts of the refractive indices, complete matching is very unlikely (Ishiji *et al.*, 2002 ▶). Nonetheless, at the Si *K* absorption edge, the reflection of the X-ray beam at the interface between Si and the soft-matter film is strongly suppressed. As a consequence, in GISAXS the signal from this interface has only a minor contribution, which may be the case due to the low surface roughness of Si. An example comparing GISAXS at standard high X-ray photon energies and at energies around the Si *K* absorption edge is shown in Fig. 9 ▶. Okuda and co-workers investigated polystyrene-*block*-poly(ethylenebutyl­ene)-*block*-polystyrene (SEBS) triblock copolymer films on Si substrates (Okuda *et al.*, 2012 ▶). In Fig. 9 ▶(*b*), the two-dimensional GISAXS pattern measured at 12.4 keV at an incident angle of 0.15° is shown. It exhibits the same features in the Yoneda peak region as for soft X-rays with a photon energy of 1.770 keV measured at an incident angle of 0.75° (see Fig. 9 ▶ *c*). A Yoneda peak at the critical angle of Si and of SEBS is present in both sets of data. In the two-dimensional GISAXS pattern measured at 1.837 keV, which is just below the absorption edge, this feature disappears and instead only one Yoneda peak is seen (see Fig. 9 ▶ *d*), since the contrast between the real parts of the refractive indices of the polymer and the Si substrate vanishes (Okuda *et al.*, 2012 ▶).

Depending on the system under investigation, the use of X-ray photon energies at other absorption edges can be advantageous as well. For example, GISAXS at the *K* absorption edge of phosphorus (2.143 keV) has been used to study phospholipid alloy films spin cast on Si substrates (Okuda *et al.*, 2014 ▶). With photon energies between 2.11 and 2.145 keV, which are just below and at the *K* absorption edge of phosphorous, anomalous grazing-incidence small-angle X-ray scattering (AGISAXS) measurements were performed by analogy with anomalous SAXS (ASAXS) measurements at a multitude of different X-ray photon energies. One should note that the term ‘anomalous scattering’ was originally used in the hard X-ray energy regime and might be unconventional for soft X-rays.

At even lower X-ray photon energies, interesting absorption *K* edges of soft-matter materials are located, such as the oxygen (543 eV), nitrogen (409 eV) and carbon (284 eV) *K* edges. The fine structure of the absorption edge can be utilized in GISAXS measurements as well. Similar to the transmission analogue, resonant soft X-ray scattering (RSoXS), the so-called grazing-incidence resonant soft X-ray scattering (GI-RSoXS) makes use of this idea. RSoXS has already been applied successfully to many different soft-matter systems (Wang *et al.*, 2009 ▶, 2011 ▶; Swaraj *et al.*, 2010 ▶; Chen *et al.*, 2011 ▶), whereas GI-RSoXS studies have only recently been performed (Ruderer *et al.*, 2013 ▶). As an initial step, the near-edge X-ray absorption fine structure (NEXAFS) spectrum needs to be probed for the soft-matter sample under investigation. Although for many polymers a large database of NEXAFS spectra is available, *e.g.* the Ade database (Watts *et al.*, 2011 ▶), deviations in the details might arise from different sources of material, as well as from the slight energy calibration differences across different facilities (Ruderer *et al.*, 2013 ▶). Typically, the NEXAFS spectra of polymers, which have low contrast in the real part of the refractive index in the hard photon energy regime, show significant differences in the soft X-ray regime. For example, in the polymer blend of P3HT and poly[5-(2-ethylhexyloxy)-2-methoxycyanoterephthalylidene] (MEH-CN-PPV), contrast can be maximized by making use of an X-ray photon energy (286 eV) at which the real part of the refractive index of P3HT is positive and that of MEH-CN-PPV is negative (Ruderer *et al.*, 2013 ▶). At this maximized contrast condition, information about the film morphology can be gained which might be inaccessible at low contrasts. Nonetheless, one should note that, for negative values of the real part of the refractive index, no total external reflection can occur and consequently no Yoneda peak can arise. Thus, two-dimensional GI-RSoXS data can look very different to GISAXS patterns measured at hard X-ray photon energies.

**Figure 10:**
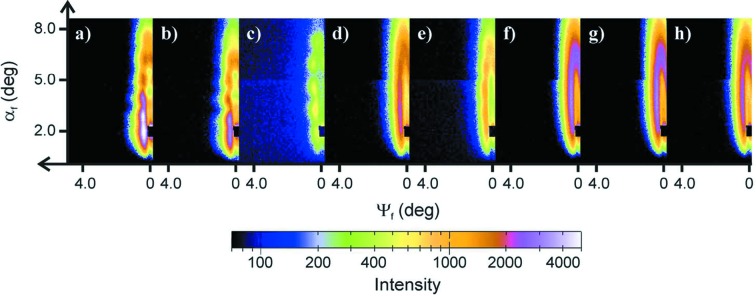
Two-dimensional GI-RSoXS data of an as-spun P3HT:MEH-CN-PPV film with a P3HT content of 70 wt%, as a function of X-ray energy, which goes from (*a*) 282 eV to (*h*) 289 eV in steps of 1 eV (left to right). The two-dimensional GI-RSoXS data are composites of two measurements with two different detector positions. The specular reflection is shielded with a beam stop. The same color coding is used for all two-dimensional data. Reprinted with permission from Ruderer *et al.* (2013 ▶). Copyright (2013) American Chemical Society.

In addition, the penetration depth of the X-ray beam, which is the depth at which the X-rays have decreased in intensity to 1/*e* of the initial intensity (Dosch, 1992 ▶), is drastically affected by the change in X-ray photon energy across the absorption edge (Okuda *et al.*, 2011 ▶). For example, at X-ray photon energies below 284 eV, the penetration depth for P3HT and MEH-CN-PPV is between 45 and 60 nm, respectively, and it decreases below 20 nm at higher energies (Ruderer *et al.*, 2013 ▶). Thus, GI-RSoXS at different energies yields structural information from different depths of the sample. As shown by Ruderer and co-workers, in the case of thin P3HT:MEH-CN-PPV blend films the surface- and volume-sensitive structure information was probed (Ruderer *et al.*, 2013 ▶). Fig. 10 ▶ shows two-dimensional GI-RSoXS data of as-spun P3HT:MEH-CN-PPV bulk heterojunction films with a P3HT content of 70 wt% (Ruderer *et al.*, 2013 ▶). The X-ray photon energy was varied from 280 to 289 eV (corresponding to a wavelength variation from 4.29 to 4.43 nm). Although the energy, and accordingly the wavelength, were only varied by 3%, the scattering data changed dramatically due to the change in penetration depth. For X-ray photon energies below 284 eV, the full thin film was penetrated and, due to roughness correlation between the polymer–substrate and polymer–vacuum interfaces, an intensity oscillation along the vertical direction was found in the two-dimensional GI-RSoXS data (see Figs. 10 ▶ *a*–10 ▶ *c*). At higher energies (above 284 eV), only Bragg-rod shaped intensity was observed, since at a low penetration depth only the surface structure is accessed (see Figs. 10 ▶ *d*–10 ▶ *h*). The changes in the total scattered intensity were attributed to the changed contrast conditions from the contrast variation explained above.

Another great advantage of NEXAFS studies, namely the angle dependence of the X-ray absorption, has great potential in GI-RSoXS, in particular in the case of wide-angle scattering experiments. The orientation of resonant bonds, *e.g.* π* antibonding orbitals that may have a particular orientation with respect to the substrate surface, causes deviations in the NEXAFS spectrum due to dipole selection rules. Since the synchrotron radiation used has a natural polarization, such GI-RSoXS experiments will be highly interesting in the field of organic electronics, which makes use of conjugated mol­ecules and polymers to a high degree. Depending on the application, *e.g.* in OLEDs, OFETs, OSCs or OPDs, different orientations of the crystalline regions are required and these can be probed with higher sensitivity compared with GIWAXS investigations.

### 2.6. GISANS and TOF-GISANS   

Similar to the X-ray based technique GISAXS, grazing-incidence scattering experiments can be performed with neutrons. For the corresponding technique, the name grazing-incidence small-angle neutron scattering (GISANS) has been established. Differences between the two techniques, GISAXS and GISANS, arise from the different scattering mechanisms, which in turn cause different contrasts. In particular, the possibility of enhancing contrast by deuteration (exchange of hydrogen by deuterium) and the possibility of seeing hydrogen render GISANS an interesting alternative to GISAXS for soft-matter samples (Müller-Buschbaum, 2013 ▶). Both small molecules and polymers can be deuterated, which significantly alters the neutron scattering length density, but has only a small effect on the X-ray scattering length density (Müller-Buschbaum, Cubitt & Petry, 2003 ▶).

Similar to GISAXS, GISANS measurements can be done at SANS instruments by integrating control of the sample position and orientation, and by using a point-shaped neutron beam. The first GISANS experiments were pioneered by Müller-Buschbaum and co-workers (Müller-Buschbaum, Gutmann & Stamm, 1999 ▶). The authors investigated the dewetting of confined polymer films and compared results from GISAXS, GISANS and AFM. Deuterated polystyrene films (dPS) were destabilized on Si substrates by thermal annealing and by solvent vapor annealing. The resulting nano-dewetting structure comprised droplets of dPS located on the Si surface at a characteristic distance. Already in these very first experiments, lateral structures well above 500 nm were resolved (in GISAXS and GISANS). The structural information delivered by all the applied techniques, GISAXS, GISANS and AFM, agreed very well due to the simplicity of the resulting morphology. However, due to the large difference in primary flux between GISAXS at a synchrotron source and GISANS at a neutron source, the counting times were significantly different. In the GISANS measurements, several hours of data acquisition time were required, whereas in the GISAXS measurements, much shorter times (minutes) gave sufficiently good statistics. Due to the strong progress in synchrotron radiation sources compared with neutron sources, this difference in required data acquisition time is significantly greater today.

The potential of the GISANS method becomes more obvious with an increase in the complexity of the sample morphology under investigation. When replacing the homopolymer dPS by a mixture of dPS and poly(*para*-methyl styrene), denoted PpMS, in the investigation of dewetted confined thin films, the droplets resulting from the dewetting exhibit an additional inner structure due to phase separation (Müller-Buschbaum, Gutmann, Cubitt & Stamm, 1999 ▶). This phase separation structure is inaccessible to GISAXS due to the very weak X-ray contrast between dPS and PpMS, and it is invisible to AFM due to the low mechanical contrast between the two polymers. In contrast, due to the deuteration, dPS and PpMS have a strong contrast for neutrons and thus only in GISANS can the inner droplet structure be observed. As a consequence, for binary systems, the contrast conditions might be more advantageous for GISANS than for GISAXS (of course GI-RSoXS might be an alternative as well). The same holds when replacing a polymer blend with a diblock co­polymer. In the diblock copolymer, the two immiscible and chemically different polymers are chemically linked together *via* a covalent bond, which turns the phase separation structure into a micro-phase separation structure. As a result, all characteristic length scales are significantly smaller in diblock copolymer morphologies and might exhibit a higher degree of order. Using GISANS and GISAXS as well as AFM, poly(styrene-*block*-*para*-methyl styrene) diblock copolymers confined within isolated droplets were investigated (Müller-Buschbaum *et al.*, 2001 ▶). Again, a dewetting mechanism was used to create these droplets on Si surfaces. Only GISANS showed the ability to probe the micro-phase separation structure inside the droplets. The observed characteristic spacing (of 72 nm) was enlarged compared with the volume micro-phase separation structure, which implies a stretching of the polymer chains parallel to the substrate surface.

In addition to the investigation of structures created by dewetting of initially stable polymer films, GISANS has turned out to be a powerful method to probe the inner film structure of homogeneous films as well. As an example, the enhanced thermal stability of thin polymer bilayer films against de­wetting was investigated with GISANS. A thermally unstable dPS layer on top of an amorphous polyamide (PA) film was stabilized by the addition of the copolymer poly(styrene-co-maleic anhydride) (SMA2), containing 2% maleic anhydride groups in the chain (Wunnicke *et al.*, 2003 ▶). Lateral structures inside the dPS:SMA2 layer were detected as a function of different annealing times. Contrast arose due to the presence of a protonated minority (SMA2) in a deuterated majority (dPS). A reversed combination of protonated and deuterated materials would have caused an unnecessarily high background due to the large incoherent scattering from hydrogen, which could easily have exceeded the weak GISANS signal. As a consequence, for GISANS measurements of (thick) homogeneous polymer films with an inner structure which should be detected, it is necessary to reduce background as much as possible, which includes incoherent scattering in addition to scattering from windows or short air paths in the setup.

**Figure 11:**
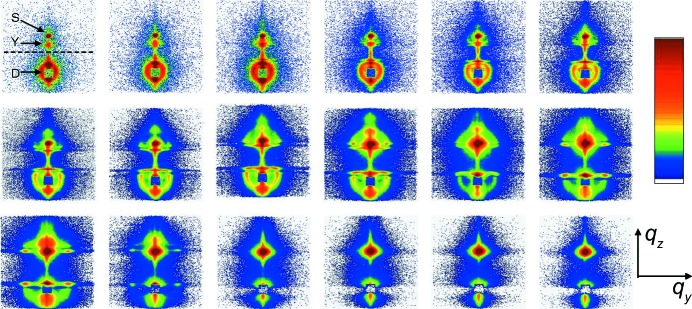
Two-dimensional GISANS data measured simultaneously in a TOF-GISANS experiment. From top left to bottom right, the corresponding mean wavelengths are 0.25, 0.28, 0.30, 0.33, 0.37, 0.40, 0.44, 0.48, 0.53, 0.58, 0.64, 0.71, 0.77, 0.85, 0.93, 1.02, 1.12 and 1.23 nm. The intensities are shown on a logarithmic scale, as indicated by the color bar. Each image covers a different range in (*q*_*y*_, *q*_*z*_) space, as explained in the text. In the top left two-dimensional image the sample horizon (dashed line), the specular peak (S), the Yoneda peak (Y) and the direct beam position (D) are indicated. Adapted from Müller-Buschbaum *et al.* (2014 ▶).

Using time-of-flight (TOF)-GISANS, a highly interesting alternative to fixed-wavelength GISANS has been developed recently (Müller-Buschbaum *et al.*, 2009 ▶). In TOF-GISANS, a broad neutron wavelength band is used instead of a fixed neutron wavelength. At each neutron wavelength from the wavelength band, a single GISANS data set is obtained simultaneously. As a consequence, a range of different scattering vectors are directly probed in TOF-GISANS by a single measurement under a fixed single angle of incidence. Moreover, the penetration depth varies with neutron wavelength. Therefore, with an optimized incident angle of the neutron beam, this translates into one part of GISANS measurements which exhibits surface sensitivity and one part which has bulk sensitivity. Fig. 11 ▶ shows the rich two-dimensional GISANS patterns which can be gained from complex morphologies, such as the micro-phase separation structure of diblock copolymers, using TOF-GISANS (Müller-Buschbaum *et al.*, 2014 ▶). A defect-rich polystyrene-*block*-(deuterated poly­methyl­methacrylate) film was investigated. In contrast with typical GISAXS patterns, the GISANS data include information below the sample horizon (dashed line in Fig. 11 ▶), which originates from transmission scattering under the shallow incident angle. Typically, the direct beam needs to be shielded, whereas the specular beam and the Yoneda peak are probed with the two-dimensional detector. With an increase in neutron wavelength, the Yoneda peak moves upwards in its position on the detector and coincides with the specular beam at the critical value of the *z* component of the scattering vector (Fig. 11 ▶, bottom row, third image from the left). At longer neutron wavelengths only surface-sensitive structural information is probed.

So far, TOF-GISANS has been used to study, for example, the dewetting of thin polymer films (Müller-Buschbaum *et al.*, 2009 ▶), the infiltration of conjugated conducting polymers into titania network structures for application in hybrid solar cells (Kaune *et al.*, 2010 ▶; Rawolle *et al.*, 2013 ▶), the micro-phase separation structure inside diblock copolymer films (Busch *et al.*, 2011 ▶; Metwalli *et al.*, 2011 ▶; Müller-Buschbaum *et al.*, 2014 ▶) and the self-assembly of diblock copolymer–maghemite nanoparticle hybrid thin films (Yao *et al.*, 2014 ▶). At the moment, neutron sources have limited neutron flux which spreads into a broad wavelength band, requiring long counting times of several hours to achieve reasonable statistics, thus limiting the use of TOF-GISANS. However, with new upcoming neutron sources, such as the European Spallation Source (ESS) in Lund, the possibilities of TOF-GISANS will be greatly extended and kinetic studies will become accessible as well.

## 3. Summary and conclusions   

Techniques related to advanced grazing-incidence scattering have benefited greatly from progress in software development and instrumentation. As a result, in addition to common GISAXS, GIWAXS, GISANS and GIWANS experiments, recently developed options and derivatives now exist, allowing the study of samples which were inaccessible in the past. Using the novel methods which have emerged during the last few years, an in-depth morphological characterization of even very complex soft-matter samples is possible. Therefore, custom-made samples are no longer required for scattering experiments. The problems related to very small samples or the need for a local scattering experiment have been overcome with the use of micro- and nanobeam GISAXS and GIWAXS. Kinetic information about morphology development during film preparation *via* a multitude of different coating techniques, as well as during transitions induced by external fields, has become accessible *via in situ* GISAXS and GIWAXS studies. Soft-matter samples in action, meaning under realistic operating conditions, such as in catalysis, sensors or devices, can be studied with *in operando* GISAXS and GIWAXS. Sample systems which impose challenges due to limited contrast in the commonly used hard X-ray photon energy regime can be probed by either soft X-rays (GI-RSoXS) or with neutrons (GISANS and GIWANS). Making use of the polarization of the X-ray beam at a synchrotron radiation source, GI-RSoXS may become an even more exciting tool in the future. TOF-GISANS opens possibilities which are difficult to access in the X-ray regime due to the risk of radiation damage when a broad X-ray wavelength band of high intensity would be used.

Beside these different aspects, which have been discussed in more detail, additional options for grazing-incidence techniques exist which we have not deepened in this article. Among them are grazing-incidence ultra-small angle X-ray scattering (GIUSAXS) experiments, which are an analogue to USAXS experiments in the transmission geometry and have proven to be able to access structures above 10 µm (Müller-Buschbaum *et al.*, 2007 ▶), and GISAXS tomography, which combines standard tomography ideas with the potential of GISAXS and thereby allows one to overcome the footprint effect for very local scattering experiments (Kuhlmann *et al.*, 2009 ▶). With the arrival of fully coherent sources, more and more coherent GISAXS will emerge, allowing the combination of X-ray photon correlation spectroscopy with GISAXS, as well as developing phase retrieval methods.

In terms of data analysis, in addition to the in-house analysis codes which have implemented the DWBA for simulating GISAXS/GISANS data, a variety of different and sophisticated software packages are available. Without the need for developing one’s own analysis software, the use of these packages will contribute significantly to a more in-depth analysis of the data, as well as allowing new groups to use advanced grazing-incidence techniques for their modern soft-matter materials analysis.

As a consequence of all the reported new options, the authors are convinced that the use of GISAXS, GIWAXS, GISANS and GIWANS in all their different facets will broaden in the future. In addition, novel derivatives of these techniques will be developed, which in turn will further extend the capabilities.
